# 

**Published:** 1888-04-07

**Authors:** 


CONTENTS.

**London Hospitals and Metropolitan Members** 1

**Words of Consolation.** Chapter LXXII.—The Sufferings of Christ 2

**Four Worlds** 2

**The Doctor's Pulpit.**—Nerve-Storms 3

**Lady Dufferin's Fund** 4

**Aids to Nursing.** By M. M. Basil, M.A., M.B., Author of "The Commoner Accidents to Life and Limb."—Food and Feeding 5

**Scotch Physicians and Surgeons.** By C. G. Furley.—Sir Robert Christison 6

**The Open-Door System** 7

**Notes and News** 8

**Everybody's Page** 10

**Annotations.**—The Presidency of the College of Physicians.—Infectious Cases: Temporary or Permanent Hospitals.—Mr. Walter Besant's Craze about Women 11

**A Temple of Lucifer** 12

**An Ishmaelite.** By Leslie Keith, Author of "The Chilcotes," "East and West," etc. Chapter I.—Father and Son 13

**The Nursing Mirror.**—The British Nurses Association.—A Plea for the Nurses.—Explanations and Suggestions.—Nursing Home at Cairo 15

**Amusements with Profit for all Ages.**—For Men and Women.—Children's Corner.—Puzzles, etc. 16

